# Genome Sequences of *Streptomyces griseus* Phages Destructrice and Rikishi

**DOI:** 10.17912/micropub.biology.001954

**Published:** 2026-04-07

**Authors:** Elizabeth A. Powell, Allison O. Wortman, Kathryn E. Tyler, Karina Alfonso-Perez, Giselle A. Bez, Caroline R. Buechlein, Michael D. Day, Tasline T. Diab, Isabella G. Henderson, Tosin E. Ishola, Ross S. Rider, Dalton N. Seiler, Colin S. Stooksberry, Allyson E. Wary, Julie A. Merkle

**Affiliations:** 1 Biology, University of Evansville, Evansville, Indiana, United States; 2 Biology, Northwest Nazarene University, Nampa, Idaho, United States

## Abstract

This paper describes the complete genome sequences of two novel bacteriophages isolated using *Streptomyces griseus* . Destructrice is a podovirus bacteriophage with a 45,548 bp genome assigned to the actinobacteriophage cluster BF. In contrast, Rikishi is a cluster BE2 siphovirus with a 132,567 bp genome.

**Figure 1. Streptomyces griseus phages Destructrice and Rikishi virion and plaque morphology:** Plaques formed by Destructrice (A) and Rikishi (C) on plates spread with *Streptomyces griseus* . Black arrows indicate plaques for each bacteriophage. Destructrice produces medium-sized, clear plaques (1.70 ± 0.397 mm), while Rikishi produces smaller, clear plaques (0.83 ± 0.322 mm). Transmission electron micrographs of Destructrice (B) and Rikishi (D) stained with 1% uranyl acetate and viewed at 100 kV accelerating potential in a JEOL 1400Plus transmission electron microscope (Western Kentucky University Southern Kentucky Center for Advanced Microscopy). Phage dimensions are provided in Table 1.

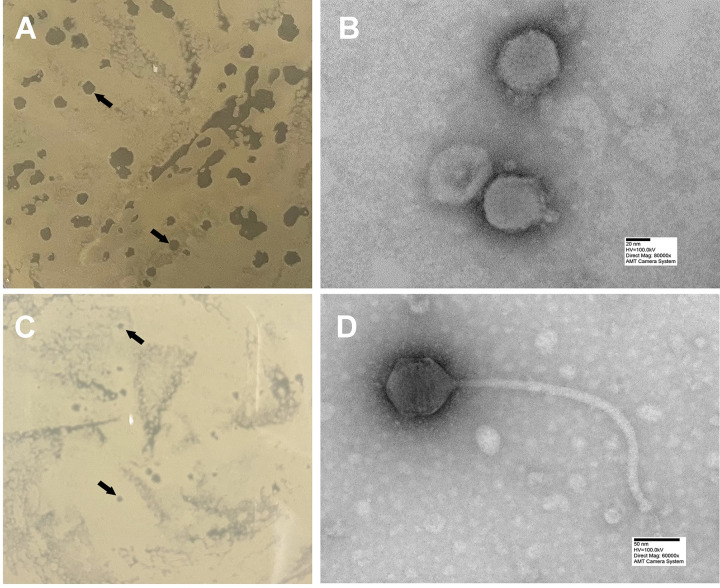



## Description

Lytic phage cocktails have been used to successfully treat antibiotic-resistant bacterial infections in humans and animals (Cristobal-Cueto et al., 2021; Hatfull et al., 2022). They have also been used as biocontrol agents in the food-processing, agriculture, and aquaculture industries (Cristobal-Cueto et al., 2021). To support these applications, isolating and characterizing novel phages that infect different bacterial species can help to diversify the library of known phages. Here we introduce two bacteriophages that infect *Streptomyces griseus* : Destructrice and Rikishi.

Destructrice and Rikishi were isolated from moist soil samples collected in Kentucky and Illinois in September 2024 (Table 1). Enriched isolation, purification, and amplification were performed using standard protocols (Zorawik et al., 2024). Briefly, soil samples were suspended in nutrient broth (Research Products International) supplemented with MgCl _2_ , Ca(NO3) _2_ , and dextrose. Soil suspensions were centrifuged, the supernatant was filtered (0.22-µm filter) and the filtrate seeded with *Streptomyces griseus* (ATCC 10137) and incubated for 3 days at 20°C. The resulting enriched culture was filtered, and the filtrate plated with *Streptomyces griseus* onto nutrient agar (Research Products International) supplemented with MgCl _2_ , Ca(NO3) _2_ , and dextrose, and was incubated for 2 days at 30°C. Two rounds of purification were performed by picking individual plaques and replating, after which a high-titer lysate was prepared. Destructrice and Rikishi both produced clear plaques, with Destructrice plaques 1.70 ± 0.997 mm in diameter (n= 86) and Rikishi plaques 0.83 ± 0.322 mm (n=27) (Figure 1). Plaques were measured using the ViralPlaque macro on Fiji (Cacciabue et al., 2019). Negative-stain transmission electron microscopy using 1% uranyl acetate showed that Destructrice has a podovirus morphology with a very short (16 ± 2 nm) tail and Rikishi a siphovirus morphology with a long (364 ± 30 nm) tail ( Figure 1, Table 1).

Phage genomic DNA was extracted from phage lysates using the Promega Wizard DNA cleanup kit. Phages were prepared for sequencing using the NEBNext Ultra II FS library preparation kit and were sequenced using the Illumina NextSeq 1000 (XLAP-P1 kit) with 100-base, single-end reads. Raw reads were trimmed with Cutadapt 4.7 (using the option: -nextseq-trim30) and filtered with Skewer 0.2.2 (using the options: -q 20 -Q 30 -n -1 50) prior to assembly (Martin, 2011; Jiang et al., 2014). Sequences were then assembled using Newbler v2.9 (Margulies et al., 2005) and Unicycler (Wick et al. 2017), and genome completeness and termini were evaluated using Consed v29 (Gordon et al., 1998; Russell, 2018). Sequencing and genome information is summarized in Table 1. Based on gene content similarity of above 35% to phages in the Actinobacteriophage database (https://phagesdb.org/), Destructrice was assigned to cluster BF and Rikishi was placed in subcluster BE2 (Pope et al., 2017; Hatfull, 2020).

Genomes were autoannotated using DNAMaster v5.23.6 (Pope and Jacobs-Sera, 2018) with Glimmer v3.02b (Delcher et al., 2007) and Genemark v4.28 (Besemer and Borodovsky, 2005). Gene calls were refined using Phamerator v589 (Cresawn et al., 2011), Starterator v589 (http://phages.wustl.edu/starterator/), and BLASTp, using the Actinobacteriophage and NCBI non-redundant database (Altschul et al., 1990), according to criteria outlined in the SEA-Phages Phage Genomics Guide (https://genomicsguide.seaphages.org/). Protein functions were determined using BLASTp, Phamerator (Actino_draft database v578), HHPred (PDB_mmCIF70, Pfam-v.36, UniProt-SwissProt-Viral70_3, and NCBI Conserved Domains databases) (Söding et al., 2005; Zimmerman et al., 2018) and Deep TMHMM v1.0 (Hallgren et al., 2022). tRNAs and tmRNAs were identified using Aragorn v1.2.41 (Laslett and Canback, 2004) and tRNAscanSE v2.0 (Lowe and Eddy, 1997). Default settings were used for all software.

Destructrice encodes 63 predicted protein-coding genes and a cassette of 21 tRNAs. Like other cluster BF phages, Destructrice has a small genome (cluster range = 44859-46511 bp), no identifiable tape measure protein (absent in annotated *Streptomyces griseus* podovirus phage), and a short terminal repeat (265 bp). Rikishi has 245 protein-coding genes, 42 tRNAs, and 1 tmRNA. Like other BE2 cluster phages, Rikishi has a large genome (cluster range = 126524-133969 bp), a long tape measure protein (6300 bp), and a long terminal repeat (12484 bp). Like other phages in clusters BE2, A, and BD, Rikishi has a DNA primase that is split into two reading frames (Cresawn et al., 2011; Russell and Hatfull, 2017). For both phage, tRNAs identified are also found in a similar location in other phage genomes from the same cluster. No integrase or immunity repressor functions were identified in either genome, suggesting lytic life cycles.

Data Availability

Destructrice is available at GenBank with Accession No. PX234433 and Sequence Read Archive (SRA) No. SRX28484007 . Rikishi is available at GenBank with Accession No. PV876926 and Sequence Read Archive (SRA) No. SRX28484029 .

TABLE 1: Properties of Streptomyces phages Destructrice and Rikishi.

**Table:** 

Bacteriophage	Destructrice	Rikishi
Location found	Louisville, Kentucky	Albion, Illinois
Location coordinates	38.20882 N, 85.75387 W	38.43662 N, 88.07433 W
Capsid diameter [nm ± SD (n)]	63 ± 3 nm (n=5)	85 ± 12 nm (n=5)
Tail length [nm ± SD (n)]	16 ± 2 nm (n=5)	364 ± 30 nm (n=5)
Genome size (bp)	45548	132567
Approximate coverage (x)	4540	1582
Number of reads	2.9 M	1.9 M
GC content (%)	59.80%	49.40%
Direct terminal repeat length	265	12484
Cluster	BF	BE2
Number of protein coding genes	63	245
Number of tRNAs	21 tRNAs	42 tRNAs and 1 tmRNA
GenBank accession no.	PX234433	PV876926
SRA accession no.	SRX28484007	SRX28484029
Isolated by	K. Tyler and P. Kocher	D. Seiler and D. Wolfe
